# Preoperative Epidural Steroid Injections and Surgical Site Infection Risk in Lumbar Spine Surgery: A Retrospective Cohort Study

**DOI:** 10.7759/cureus.84892

**Published:** 2025-05-27

**Authors:** Muhammad Waheed, Brandon Henry, Abdurrahman Ehsan, Andreea Geamanu, Chaoyang Chen, Rahul Vaidya, Anil Sethi

**Affiliations:** 1 Orthopaedic Surgery, Detroit Medical Center Wayne State University, Detroit, USA; 2 Orthopaedic Surgery, Michigan State University College of Osteopathic Medicine, East Lansing , USA; 3 Department of Biomedical Engineering, Wayne State University, Detroit , USA; 4 Orthopaedic Surgery, Wayne State University School of Medicine, Detroit, USA

**Keywords:** epidural steroid injection, lumbar fusion, lumbar spinal decompression, postoperative outcomes, surgical site infection

## Abstract

Background: Low back pain is a leading cause of work-related disability worldwide. Epidural steroid injections (ESIs) are commonly used as nonoperative treatments for patients with degenerative lumbar spine pathology. It is imperative to further elucidate the association between preoperative ESIs and postoperative outcomes in this population. This study seeks to evaluate the association between the timing of preoperative ESIs and the incidence of surgical site infections (SSIs) in patients undergoing lumbar spine surgery.

Methods: This retrospective study analyzed patients who underwent lumbar spine surgery from January 2020 to December 2021. Patients were stratified based on ESI status: no ESI, most recent ESI within three months preoperatively (early group), and most recent ESI equal to or greater than three months preoperatively (late group). All patients were followed for at least one year postoperatively to monitor for SSI. Bivariate correlation analysis using Spearman’s rank correlation coefficient was performed to identify risk factors for SSI. Infection rates across ESI groups were compared using Pearson’s chi-square test, Fisher’s exact test, or the chi-square test of independence, selected based on sample size and expected cell counts.

Results: Among 94 patients, 49 received a preoperative ESI. Of these, 11 patients were in the early ESI group and 38 in the late ESI group. The overall infection rate was 2.2%, with one infection in each ESI subgroup. No significant difference in SSI rates was observed between the early and late ESI groups (p=0.171). Higher BMI, use of Disease-Modifying Antirheumatic Drugs (DMARDs), and higher American Society of Anesthesiologists (ASA) scores were associated with an increased SSI risk (p<0.05).

Conclusions: The timing of preoperative ESI does not significantly influence SSI risk in lumbar spine surgery. This study also highlights the role of elevated BMI, DMARD use, and ASA scores as potential risk factors for SSI in patients receiving preoperative ESI. Mitigating infection risk should focus on addressing modifiable factors such as BMI, DMARD use, and ASA scores rather than ESI timing.

## Introduction

Low back pain is a prevalent and costly medical condition that is now among the leading causes of work-related disability worldwide [1,2]. Impacting over 500 million individuals and generating annual costs of up to $100 billion, the careful attention to diagnosis, treatment, and management of patients with neck and low back pain remains imperative to curtail healthcare expenses and optimize patient outcomes [3 ]. Nonoperative modalities consist of activity modification, nonsteroidal anti-inflammatory drugs, muscle relaxants, physical therapy, bracing, among others [4,5]. Spine injections, encompassing procedures such as epidural steroid injections (ESIs) and facet joint interventions, have become integral tools in the management of various spinal pathologies, offering relief to patients grappling with debilitating pain and functional limitations [6,7]. The diagnostic and oftentimes therapeutic nature of epidural spine injections make them a favorable intervention for patients suffering from neck or lower back pain [8,9]. Nearly half of patients undergoing lumbar spine surgery for stenosis or herniation have had ESIs before surgery [10]. While these injections are common, the invasive nature of these procedures introduces a conundrum: the potential vulnerability to microbial intrusion and theoretical risk for subsequent infections.

Conflicting evidence exists regarding the association between preoperative ESI and increased risk of postoperative surgical site infection (SSI) for patients undergoing spine surgery [11-16]. Some studies have reported that injections less than three months before surgery were not associated with an increased risk of postoperative SSI [13,14], while others suggest that injections within three months of surgery significantly increase the risk of SSI [12,15,16].

The objective of this study was to analyze the association between the timing of preoperative ESIs and the risk of postoperative SSIs. We hypothesize that preoperative epidural spine injections, regardless of their timing in relation to lumbar spine surgery, are not associated with an increased risk of postoperative SSIs.

## Materials and methods

A retrospective review of medical records was conducted for adult patients (aged >18 years at the time of surgery) diagnosed with lumbar stenosis or radiculopathy who underwent either lumbar spinal decompression or lumbar fusion between January 1, 2020, and December 31, 2021, at an urban academic hospital. Patients who received single or multi-level decompression with or without a fusion were included in our analysis. Exclusion criteria included patients younger than 18 years, those with a history of prior lumbar spine surgery, individuals with pathology secondary to tumor or trauma, and those undergoing revision procedures. Patients who underwent lumbosacral fusion or anterior lumbar surgery during the same hospital stay were also excluded from the analysis.

**Figure 1:**
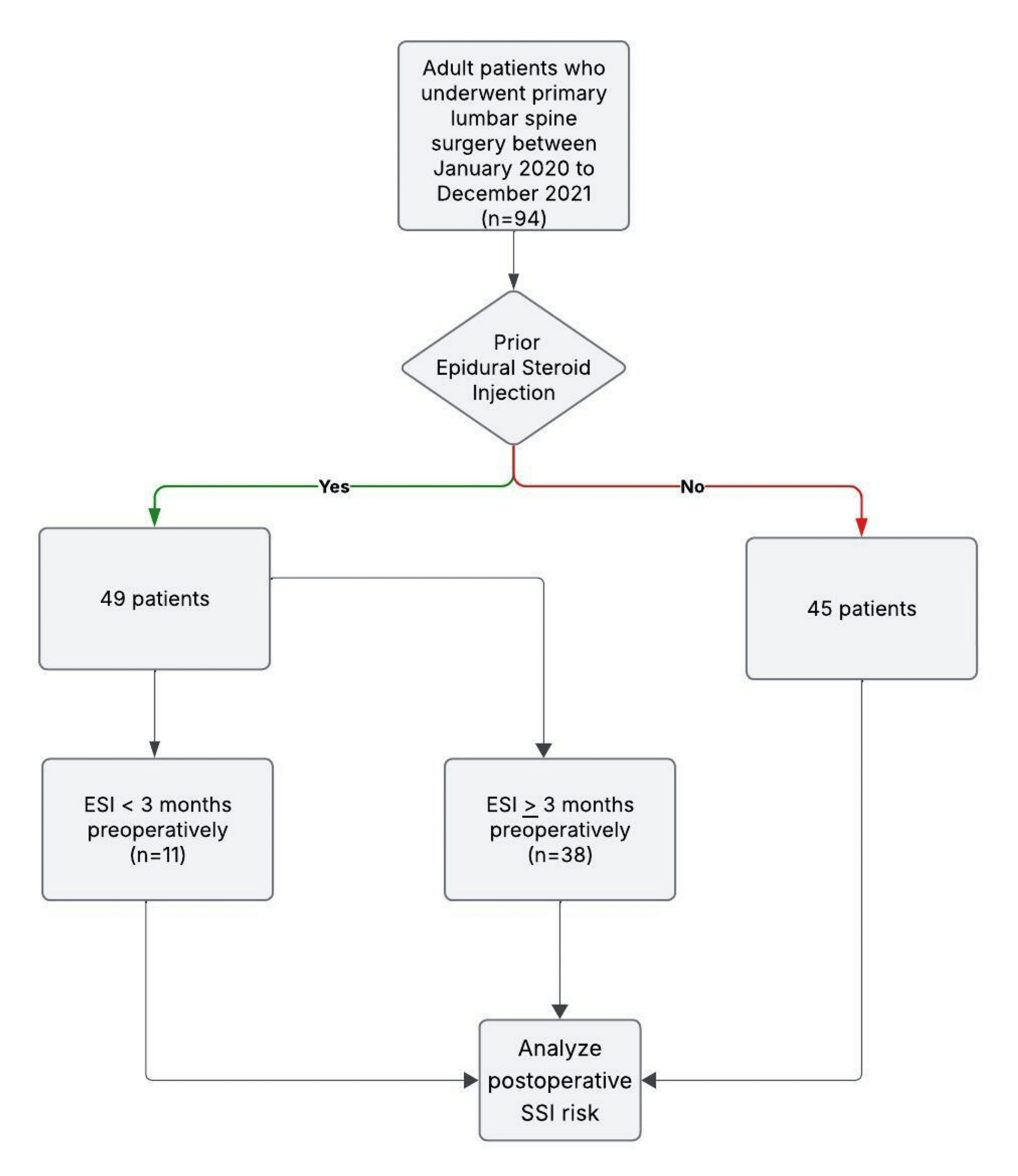
This flowchart highlights the study design and patient cohort grouping based on ESI timing ESI: Epidural steroid injection

Patient information was collected through manual medical record review within the electronic medical record. The data collection and analysis were approved by the Institutional Review Board (IRB# 23-03-5603). Patients were classified based on whether they had received an ESI prior to their lumbar spine surgery. ESIs that patients received were either transforaminal or interlaminar. They were subsequently divided into three groups based on the timing of their most recent preoperative ESI: 1) no history of ESI, 2) most recent ESI within three months preoperatively, and 3) most recent ESI greater than three months preoperatively (Figure 1). All ESIs were administered by a fellowship-trained orthopedic spine surgeon or interventional physical medicine and rehabilitation specialist.

The primary outcome of interest in this study was to explore the association between the timing of preoperative ESIs and postoperative SSIs. Various predictors and patient risk factors were also included in our analysis. The selected variables for predictors of postoperative SSI analysis included patient demographics, comorbidities, and complications. The analyzed variables included age, sex, BMI, American Society of Anesthesiologists Physical Status (ASA) score, tobacco use, disease-modifying antirheumatic drugs (DMARD) use, corticosteroids intake, blood loss amount, dural tear, and history of prior lumbar spine surgery.

Statistical analysis

Bivariate correlation analysis using Spearman’s rank correlation coefficient was performed to identify factors associated with postoperative SSI. Infection rates between treatment groups were compared using Pearson’s chi-square test, Fisher’s exact test, or the chi-square test of independence, depending on sample size and distribution. Odds ratios (ORs) and relative risks (RRs) with corresponding 95% confidence intervals were calculated to assess associations between risk factors and infection outcomes.

An independent t-test was utilized to evaluate differences in continuous variables, including age, BMI, and estimated blood loss, between the infection and non-infection groups. Multivariate analysis was conducted using factor analysis with the principal component analysis extraction method and Varimax rotation with Kaiser normalization to identify independent predictors.

Power analysis was performed using G*Power software (Version 3.1; Heinrich-Heine-University, Düsseldorf, Germany) to determine the probability of correctly rejecting a false null hypothesis. All statistical analyses were conducted using SPSS Statistics software (Version 25; IBM Corp., Armonk, NY, USA). A two-tailed P-value < 0.05 was considered statistically significant.

## Results

Among the 94 participants, 45 patients had no history of preoperative ESIs, 11 patients received an ESI within three months prior to lumbar spine surgery (early group), and 38 patients received an ESI more than three months preoperatively (late group). The mean number of injections per participant prior to surgery was 1.74 ± 0.93.

**Figure 2:**
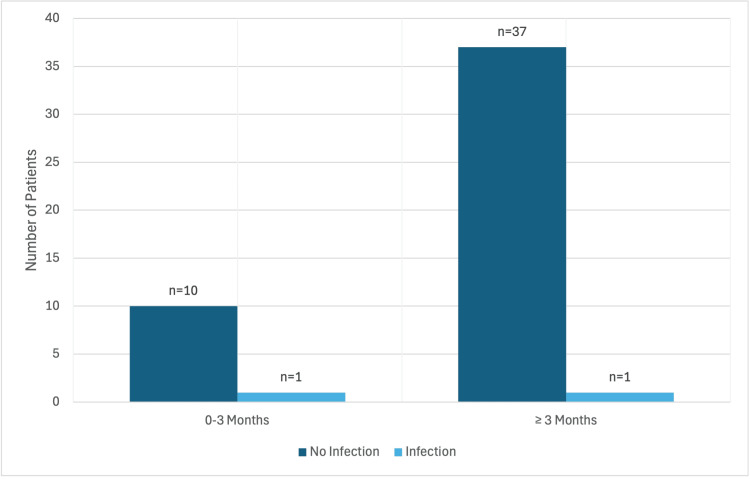
Incidence of SSIs between the early and late groups. There was no statistically significant difference between ESI timing and postoperative SSI risk between the early and late groups (p=0.171). SSI: Surgical site infection; ESI: Epidural steroid injection

Overall, two patients (2.2%) developed a postoperative SSI, with one infection occurring in each ESI subgroup (Figure 2). Bivariate correlation analysis demonstrated that postoperative infection was associated with higher BMI (correlation coefficient = 0.260, P = 0.016) and history of DMARD use (correlation coefficient = 0.392, P < 0.001).

Patients who developed infections had a significantly higher BMI (t-test, P = 0.029) and higher American Society of Anesthesiologists (ASA) scores (t-test, P = 0.001) compared to the non-infection group (Table 1). There were no significant differences between the infection and non-infection groups in terms of patient age or estimated blood loss (Table 1).

**Table 1:** A comparison of various risk factors for postoperative SSIs. Elevated BMI, ASA score, and DMARD use were significantly associated with postoperative SSIs. BMI: Body mass index; ASA: American Society of Anesthesiologists; DMARD: Disease-modifying antirheumatic drug; OR: Odds ratio; RR: Relative risk; ESI: Epidural steroid injection *Chi-Square Fisher’s exact test **Independent t-test ***Chi-Square Pearson test

Risk Factors		Surgical Site Infection		P-Value	OR	RR	Power (1-β)
		No	Yes				
Timing of ESI	No ESI	44 (100%)	0 (0%)	0.171*	–	–	77.80%
	< 3 months	10 (90.9%)	1 (9.1%)				
	> 3 months	37 (97.4%)	1 (2.6%)				
BMI		32.5± 7.0	51.2±3.4	0.029**	–	–	99.70%
Age		57.0±11.6	60.5±3.5	0.37**	–	–	8.70%
Blood loss		387.5±127.3	540.0±404.3	0.302**	–	–	10.80%
Rheumatoid arthritis	Yes	12 (93.2%)	1 (7.7%)	0.272*	–	–	95.70%
	No	75 (98.7%)	1 (1.3%)			1.724	
Corticosteroid use	Yes	3 (100%)	0 (0.0%)	0.933*	0.604		95.40%
	No	84 (97.7%)	2 (2.3%)				
DMARD use	Yes	2 (66.7%)	1 (33.3%)	0.067* 0.01***		0.046	99.90%
	No	85 (98.8%)	1 (1.2%)			1.954	
Tobacco use	Yes	65 (98.5%)	1 (1.5%)	0.452		1.494	95.50%
	No	22 (95.7%)	1 (4.3%)			0.506	
Dural tear	Yes	4 (100.0%)	0 (0.0%)	0.909*	0.952		97.10%
	No	80 (97.6%)	2 (2.4%)				
ASA		2.76±0.479	3.0±0.0	0.001**	–	–	25.40%
ESI vs no ESI preoperatively		60 (96.8%)	2 (3.2%)	0.472*	0.682		95.10%
		28 (100.0%)	0 (0.0%)				

No significant differences were observed in infection rates among the non-injection, early injection, and late injection groups (P = 0.171). Similarly, there was no significant difference in infection rates between patients with and without rheumatoid arthritis (RA). However, patients with RA who were treated with DMARDs had a significantly higher infection rate compared to RA patients not receiving DMARD therapy (chi-square test, Fisher’s exact test, P = 0.01). However, there were no statistically significant differences in infection rates based on corticosteroid use, smoking history, presence of a dural tear, or prior lumbar spine surgery (Table 1).

Factor analysis revealed that postoperative infection was strongly associated with BMI and DMARD use (P < 0.001 for both). These three variables - postoperative infection, BMI, and DMARD use - loaded onto a single component, with factor loadings of 0.774, 0.777, and 0.481, respectively.

## Discussion

ESIs have become an increasingly common nonoperative treatment option for managing spinal pathologies and alleviating pain [6,7]. Despite their effectiveness in providing short-term pain relief, the invasive nature of ESI raises concerns about the risk of infection, a potential issue highlighted in other orthopedic fields, such as arthroplasty, where preoperative injections have been associated with increased SSIs [17-19]. The literature presents conflicting findings regarding the relationship between preoperative ESIs and the risk of postoperative SSIs in spine surgery [11-16]. Some studies indicate that injections administered within three months of surgery are associated with an increased risk of infection [12,15,16]. However, other studies report no such correlation [13,14]. In this current study, we examined 94 lumbar surgery patients to assess the impact of preoperative ESI on postoperative infection rates. We found no significant difference in infection rates between the non-injection, three-month, and greater than three-month ESI groups. However, further subgroup analysis identified that higher BMI scores, DMARD use, and higher ASA scores were associated with an increased risk of SSIs.

In a recent systematic review and meta-analysis conducted by Lee et al. [11], nine studies were examined to evaluate the impact of preoperative ESIs on postoperative infection rates in lumbar spine surgery. The meta-analysis compared ESI within 30 days of surgery to controls, ESI within 30 days to ESI administered one to three months preoperatively, and any history of ESI to controls. The results revealed no significant difference in infection rates between the ESI and control groups, with rates of 2.67% for the 30-day ESI group and 1.69% for the control group, and no significant differences in other comparisons between subgroups [11]. Another study conducted by Seavey et al. examined the association of preoperative lumbar epidural corticosteroid injections on postoperative infection rates in patients undergoing single-level lumbar decompression [14]. The study analyzed 6,535 patients, including 847 who received preoperative lumbar ESI, and found no significant difference in infection rates between the injection and control groups. While the infection rate in the injection group was slightly higher (1.18% versus 0.76% in the control group), the odds ratio was not statistically significant (OR 1.57; P = 0.21) [14].

These findings suggest that preoperative ESIs do not increase the risk of postoperative infections in patients undergoing lumbar spine surgery. This aligns with our study, which also found no significant difference in infection rates between non-injection, three-month, and greater than three-month ESI groups. However, our study adds to the current literature by identifying that patients with a higher BMI, DMARD use, and ASA scores are associated with an increased risk of postoperative SSIs.

One possible explanation for this correlation is that a higher BMI, DMARD use, and higher ASA scores are linked to underlying health conditions or immune system factors that may predispose individuals to a greater risk of infection. A higher BMI is linked to obesity-related comorbidities like diabetes, which weakens the immune system, impairs wound healing, and increases the likelihood of postoperative infections [20]. DMARDs suppress the immune system to control autoimmune conditions, but this immunosuppression can make patients more susceptible to infections after surgery [21]. Additionally, a higher ASA score indicates more severe underlying health conditions, which can reduce a patient’s physiological reserves and impair their ability to heal, further increasing the risk of postoperative infection [22].

In a study carried out by Singla et al. [15], the association between preoperative lumbar ESIs and postoperative infection rates following lumbar fusion was investigated using a nationwide insurance database. The study included patients who underwent lumbar fusion with or without prior ESI, dividing them into three groups based on the timing of the injection: within one month, between one and three months, and more than three months before surgery. The results revealed a significant increase in infection risk for patients who had received ESI within one month (OR 2.6, p < 0.0001) or one to three months (OR 1.4, p = 0.0002) prior to surgery, compared to those who did not receive an injection. However, no increased risk was observed for patients who had their fusion surgery more than three months after their last ESI [15]. Likewise, another study conducted by Kreitz et al. demonstrated an increased risk of postoperative infection in patients who underwent lumbar fusion surgery following a prior ESI within one month [23].

One possible theory for the increased infection risk observed in both studies is the inclusion of lumbar fusion surgeries [24]. Fusion surgeries involve the use of instrumentation, such as rods, screws, or cages, which can act as foreign bodies and provide a surface for bacterial adhesion and biofilm formation. These factors significantly increase susceptibility to SSIs. Additionally, the instrumentation and increased tissue manipulation during fusion procedures may lead to greater intraoperative blood loss and longer operative times, both of which are established risk factors for infection [24].

Delaying surgery based on the assumption that the timing of a preoperative ESI significantly increases the risk of postoperative infections can have potential consequences. Prolonging surgical intervention may extend a patient’s pain and disability, diminishing their quality of life and potentially reducing the overall benefits of the surgery. Timely intervention is particularly crucial for conditions like lumbar disk herniation or spinal stenosis, where delays can hinder functional recovery and increase the risk of chronic complications [25,26]. Our study suggests that delaying surgery due to concerns about preoperative ESI might not be warranted, as no significant increase in postoperative infection rates was observed. Instead, focusing on modifiable risk factors, such as higher BMI, DMARD use, and ASA score, should be prioritized to improve surgical outcomes and prevent unnecessary delays.

This study has several notable limitations. As a retrospective observational analysis, it identifies associations rather than establishing causality; therefore, prospective studies or randomized controlled trials are needed to further validate or refute the relationship between preoperative ESIs and postoperative infection risk. Selection bias is also a consideration, given that all data were obtained from a single healthcare system’s electronic medical record (EMR). Although the sample size was relatively small (n = 94), a priori power analysis confirmed that the study was adequately powered to detect significant differences among the cohorts. Nevertheless, the limited sample size may limit the generalizability of the findings to broader populations. Finally, as is inherent to retrospective study designs, despite adjustment for baseline patient characteristics, the possibility of residual confounding variables influencing the outcomes cannot be entirely excluded.

## Conclusions

In this retrospective cohort study, the timing of preoperative ESIs was not associated with an increased risk of postoperative SSI following lumbar spine surgery. Although no significant difference in SSI rates was observed between patients with or without prior ESIs, higher BMI and the use of DMARDs appeared to be associated with an increased risk for SSIs. Given the relatively small sample size and retrospective design, these findings should be interpreted with caution. Further large-scale, multi-institutional prospective studies are warranted to better delineate the relationship between preoperative ESIs and postoperative SSI risk in patients undergoing lumbar spine surgery.
